# DOG1-Positive Extragastrointestinal Stromal Tumor Presenting As Large Abdomino-Pelvic Mass: A Case Report

**DOI:** 10.7759/cureus.31670

**Published:** 2022-11-19

**Authors:** Tulsi Appat, Shivani B Paruthy, Sajith K Mohan, Kashinath Singh, Anirban Das

**Affiliations:** 1 General Surgery, Vardhman Mahavir Medical College and Safdarjung Hospital, New Delhi, IND

**Keywords:** cd34, sma, cd-117, stromal tumors, dog 1, extraintestinal gist, gist

## Abstract

Gastrointestinal stromal tumors (GIST) are mesenchymal tumors commonly arising from the GI tract. Only a small number of GIST originating outside the GI tract have been reported in the literature. They are termed extraintestinal GIST (E-GIST), with histological features similar to GIST. These commonly arise from the omentum, mesentery, or abdominal wall. Microscopic examination shows spindle or epitheloid morphology with immunohistochemistry (IHC) positivity for the cluster of differentiation 117, 34 (CD117, CD34), or discovered on GIST-1 (DOG1). This case series describes the presentation of two cases of E-GIST as an abdominopelvic mass with DOG1 positivity and CD117 negativity on IHC. Patient in the first case presented with a giant abdominopelvic mass, clinically arising from the pelvis with a misdiagnosis of midline desmoid tumor. It was completely excised with a histological surprise of E-GIST with DOG1 positivity on IHC. The second case presented a swelling in the groin region, separate from the testis but arising from the anterior abdominal wall, with histological features of E-GIST with DOG1 positivity. The cases reported here show further evidence regarding the existence of a distinct subset of GISTs characterized by extraintestinal localization, with negative immunohistochemical expression of receptor tyrosine kinases (KIT) and positive DOG1 expression, which appears to be rare and makes DOG1 an emerging marker for GIST.

## Introduction

Gastrointestinal stromal tumors (GISTs) are the commonest mesenchymal tumors of the GI tract, arising from the interstitial cells of Cajal [1]. These tumors vary in their presentation, from being asymptomatic to presenting with large masses accompanied by pain and hematemesis. Such tumors arising from structures other than the GI tract with the same radiological and histopathological features as that of GIST are termed extraintestinal GIST (E-GIST). The workup and treatment of E-GIST are similar to those of GIST. The patient undergoes radiological evaluation, mostly ultrasound, followed by multi-detector computed tomography (MDCT) and tissue biopsy. The commonly used markers are receptor tyrosine kinases (KIT/CD117) immunohistochemistry and platelet-derived growth factor receptor alpha (KIT/PDGFRA) mutation analysis, which helps in planning treatment.

Approximately 90-95% of GISTs show positive staining for CD-117, but if the tumor is negative for CD117, CD34, and S-100, a definitive diagnosis is often challenging [2]. Recently, discovered on GIST-1 (DOG1), a newly introduced immunohistochemical marker and a chloride channel protein, has received considerable attention as an essential marker for the diagnosis of GIST. The tumor is resistant to radiotherapy and chemotherapy and is managed by complete surgical resection [2]. Often in non-resectable tumors or those with a high risk of malignant potential, the treatment is with tyrosine kinase inhibitors (often imatinib or, in resistant cases, sunitinib). This distinct variant of GIST usually shows KIT mutation; however, E-GIST with DOG1 expression appears to be rare. We report two cases of E-GIST arising from the anterior abdominal wall with DOG1 mutation.

## Case presentation

Case 1

**Figure 1:**
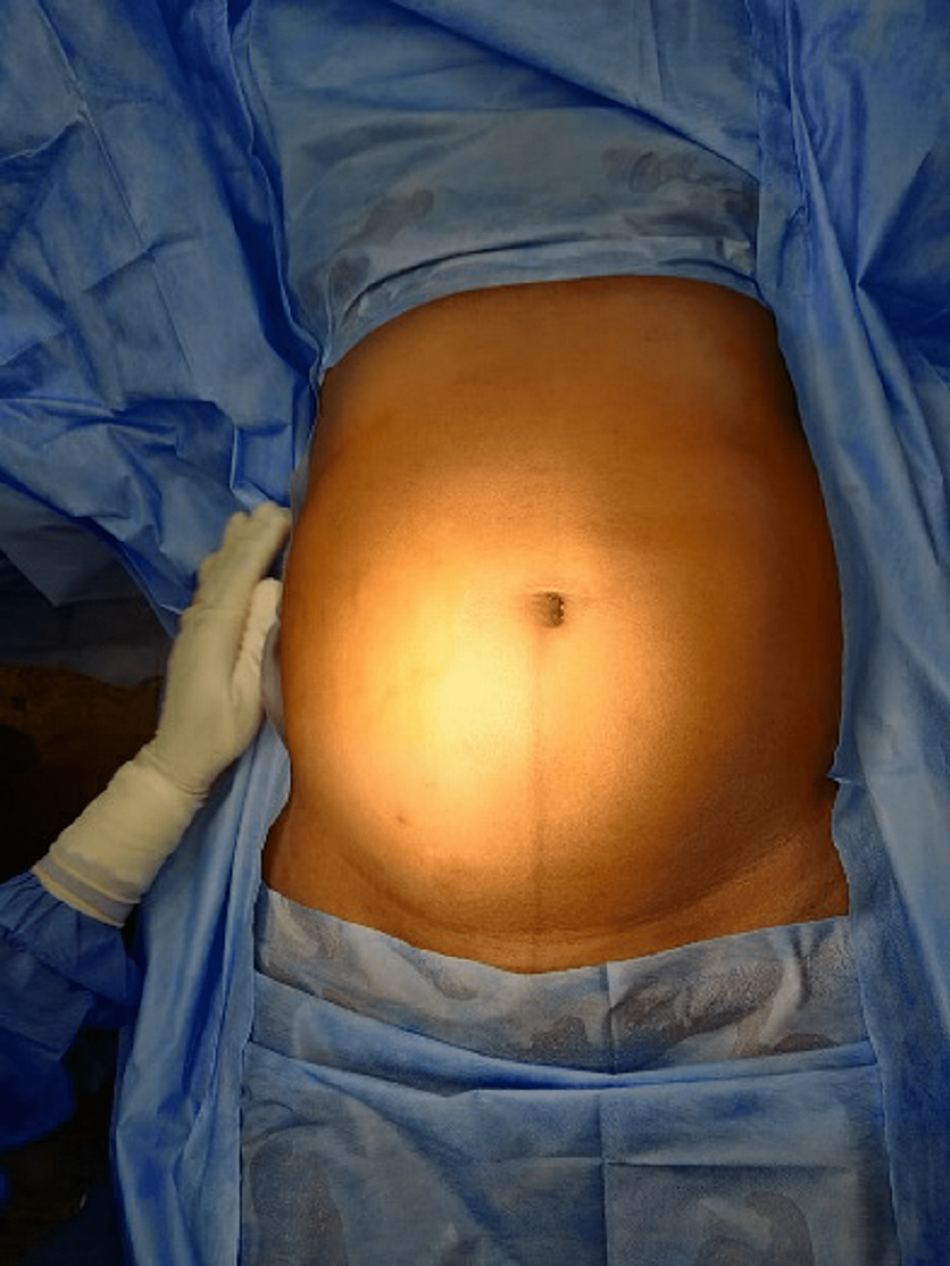
Preoperative appearance of the abdomen.

A 40-year-old female presented to the surgical clinics with complaints of a lump in her lower abdomen noticed one year ago. It started initially in the right lower abdomen of size 5 x 4 cm, then gradually progressed to a size of 15 x 15 cm involving the whole lower abdomen. Besides its huge size, the lump was mobile with posture change. Associated symptoms included dull aching pain, abdominal discomfort, and pressure symptoms on the bladder, adding to the increased frequency of micturition. Vomiting, altered bowel habits, fever, blood in stools, menstrual irregularities, and weight loss were absent. Clinical examination revealed a parietal (extra-peritoneal) lump of size approximately 15 x 15 cm involving hypochondrium, umbilical region, and reaching up to bilateral iliac fossa, with well-defined margins, smooth surface, firm to hard in consistency, non-tender, and dull on percussion (Figure 1).

**Figure 2:**
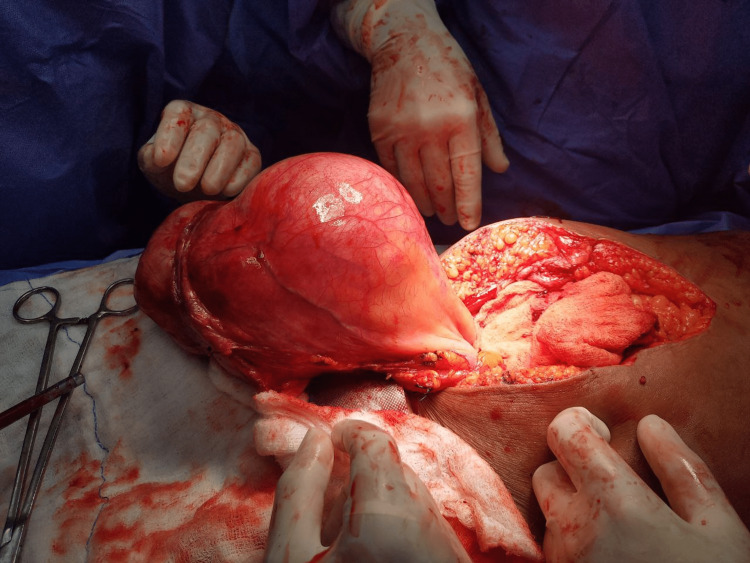
Intraoperative appearance of lump.

On USG, a heteroechoic mass lesion of approximately 15.2 x 13.0 cm with internal vascularity was seen in the lower abdomen. Incidental finding of cholelithiasis was noted. Contrast-enhanced CT of the abdomen revealed a large, well-circumscribed, heterogeneously enhancing mass of size 14 x 15 x 13.4 cm extending from the L2 vertebral body to sacral promontory, abutting and displacing multiple jejunal loops with its posterior surface. Anteriorly the mass was abutting the anterolateral abdominal wall with loss of fat planes displacing the caecum superiorly and urinary bladder inferiorly. The mass was also abutting the right ureter, right psoas muscles, and distal-most portion of the inferior vena cava with maintained fat planes. Urinary bladder, uterus, bilateral ovaries, and visualized bowel loops appeared normal. The features were suggestive more likely of GIST and less likely of a tubo-ovarian mass or desmoid tumor. Carcinoembryonic antigen (CEA) and carbohydrate antigen (CA)-125 levels were normal. The core-biopsy result was suggestive of fibromatosis or smooth muscle tumor of uncertain malignant potential. On laparotomy, there was a large well-circumscribed encapsulated extra-peritoneal mass of size 15 x 16 x 14cm, arising from the posterior layer of the anterior abdominal wall occupying supra-vesical space (Figure 2).

**Figure 3:**
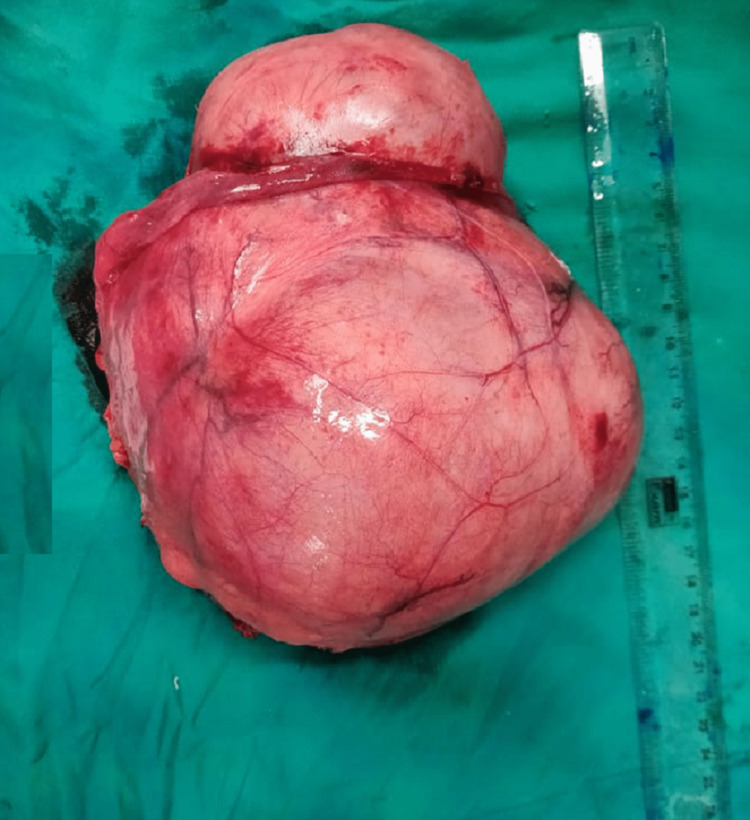
Post-excision specimen.

It was abutting the urinary bladder with maintained fat planes and adherent to the anterior abdominal wall in the infraumbilical region. The mass displaced the bowel loops superiorly and posteriorly. Bilateral ovaries, fallopian tubes, uterus, bowel loops, and the rest of the abdominal viscera were grossly normal. The gall bladder was distended with multiple black pigmented calculi, and a short cystic duct was noted. Complete excision of the mass with cholecystectomy was done. Grossly the specimen was a large encapsulated multi-lobulated mass of size 15 x 16 x 14 cm with an intact capsule with no infiltration (Figure 3).

**Figure 4:**
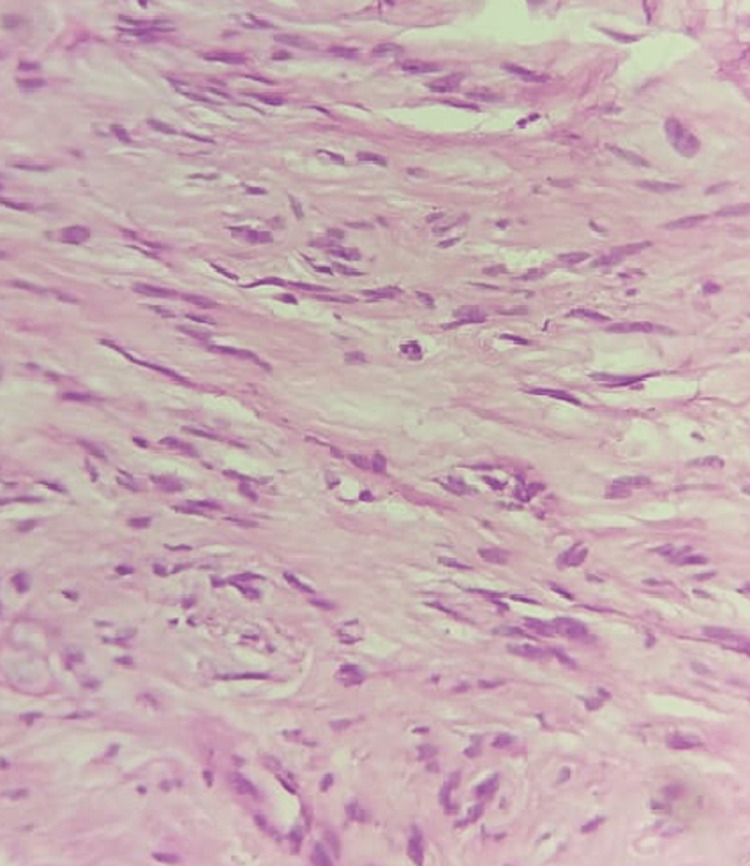
H&E staining of the specimen showing epitheliod cells.

The cut surface was grey-white firm with mucoid areas. A microscopic examination of the tumor showed spindle-shaped cells arranged in fascicles and sheets. The cells showed eosinophilic cytoplasm with elongated nucleus, foci of hyalinization, and mitotic count of 0-1/HPF (Figure 4). On IHC, the tumor showed positivity for DOG1, vimentin, desmin, and smooth muscle actin (SMA) but was negative for CD117 and CD34. The features were suggestive of GIST with myogenic differentiation. Specimen of gall bladder showed chronic cholecystitis with pigmented cholelithiasis. The patient was discharged on the fifth day, and three monthly follow-ups were done for one year. Imatinib therapy was started, and a repeat CT scan after one year showed no features of recurrence of the disease.

Case 2

**Figure 5:**
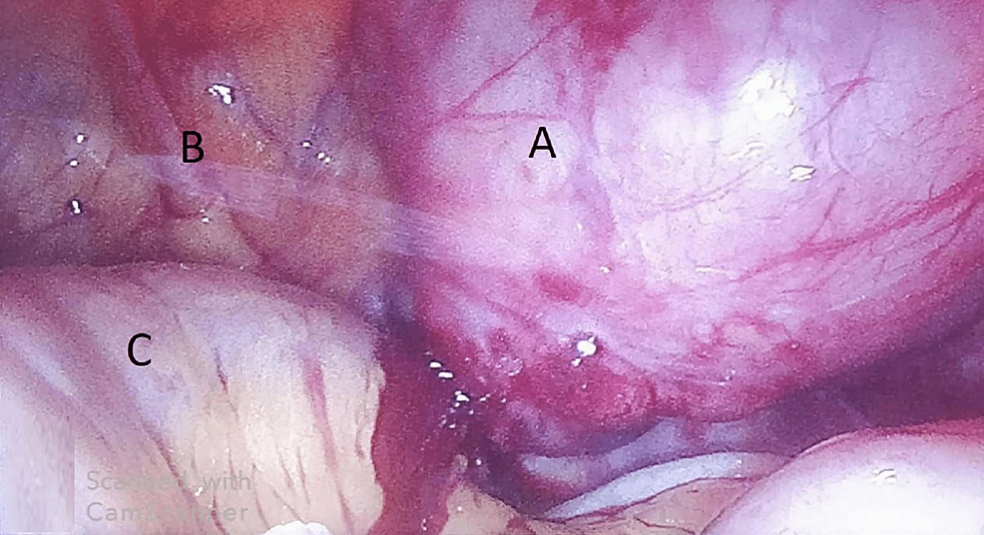
Diagnostic laparoscopic image showing the tumor arising from the parietal wall. A: Tumor, B: Parietal wall, C: Bowel loop.

A 42-year-old gentleman presented to the surgery outpatient department with right groin swelling for four months. The lump was initially in the right inguinal region and then gradually progressed to the right lower abdomen. It was prominent on standing or straining and reduced on lying down, associated with mild abdominal and pelvic discomfort. There was no history of trauma, fever, urinary complaints, altered bowel habits, previous surgeries, or other chronic comorbidities. Clinical examination in a standing position showed a well-defined, firm, non-tender, right inguinal lump of size 7 x 6 cm, with a smooth surface extending to the right iliac fossa. In the supine position, the lump was palpable in the hypogastrium and right iliac fossa, becoming less prominent on the straight leg raise test. Per rectal examination was unremarkable. Ultrasound revealed a hetero-echoic mass of size 6 x 5 cm arising from the anterior abdominal wall in the right iliac fossa region with no bowel or other organ involvement. CT abdomen showed a pedunculated mass of size 6 x 6 cm arising from the anterior abdominal wall in the right iliac fossa, with no bowel or organ involvement. The urinary bladder, rectum, ureter, and psoas muscles were normal, and mild free fluid was noted in the pelvis. Laparoscopy revealed a well-defined, lobulated, pedunculated mass of size 7 x 6 cm arising from the posterior surface of the anterior abdominal wall with a narrow pedicle, extending into the peritoneum compressing the bowel loops, with no organ involvement (Figure 5).

**Figure 6:**
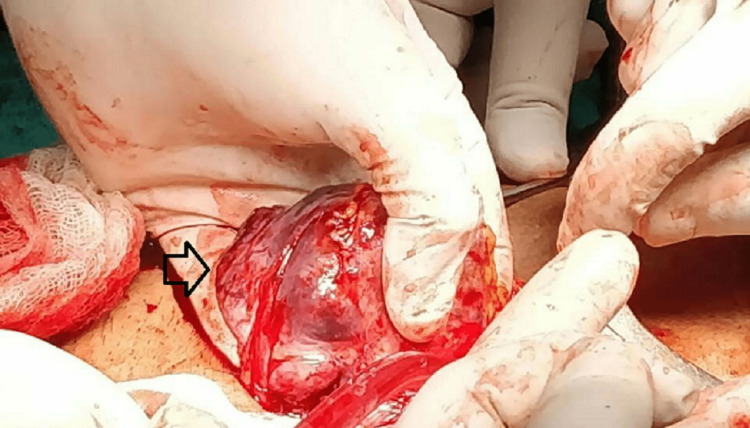
Excision of the tumor. The arrow shows the tumor.

The urinary bladder, rectum, bowel loops, and other viscera were healthy. Wide local excision of the mass with primary repair of the defect was done (Figure 6).

**Figure 7:**
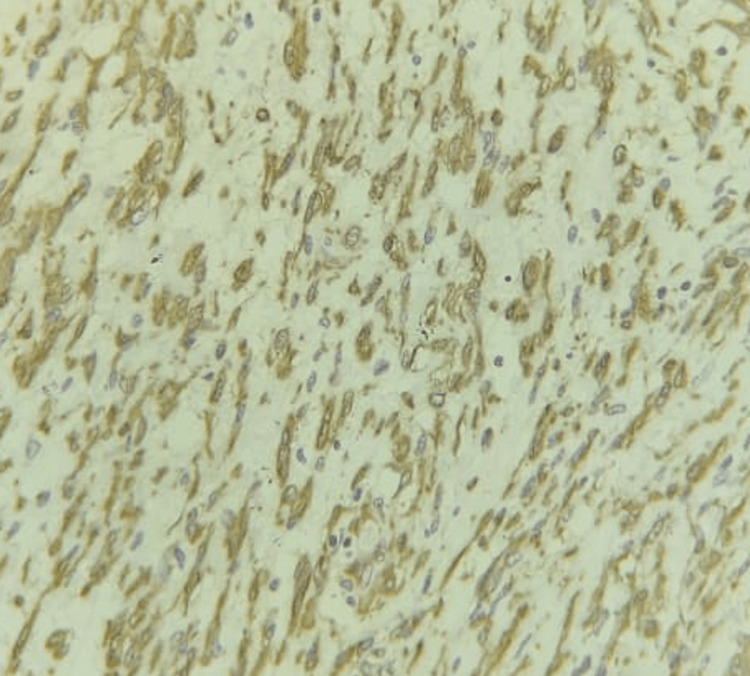
IHC of the specimen showing SMA positivity. IHC: Immunohistochemistry; SMA: Smooth muscle actin.

**Figure 8:**
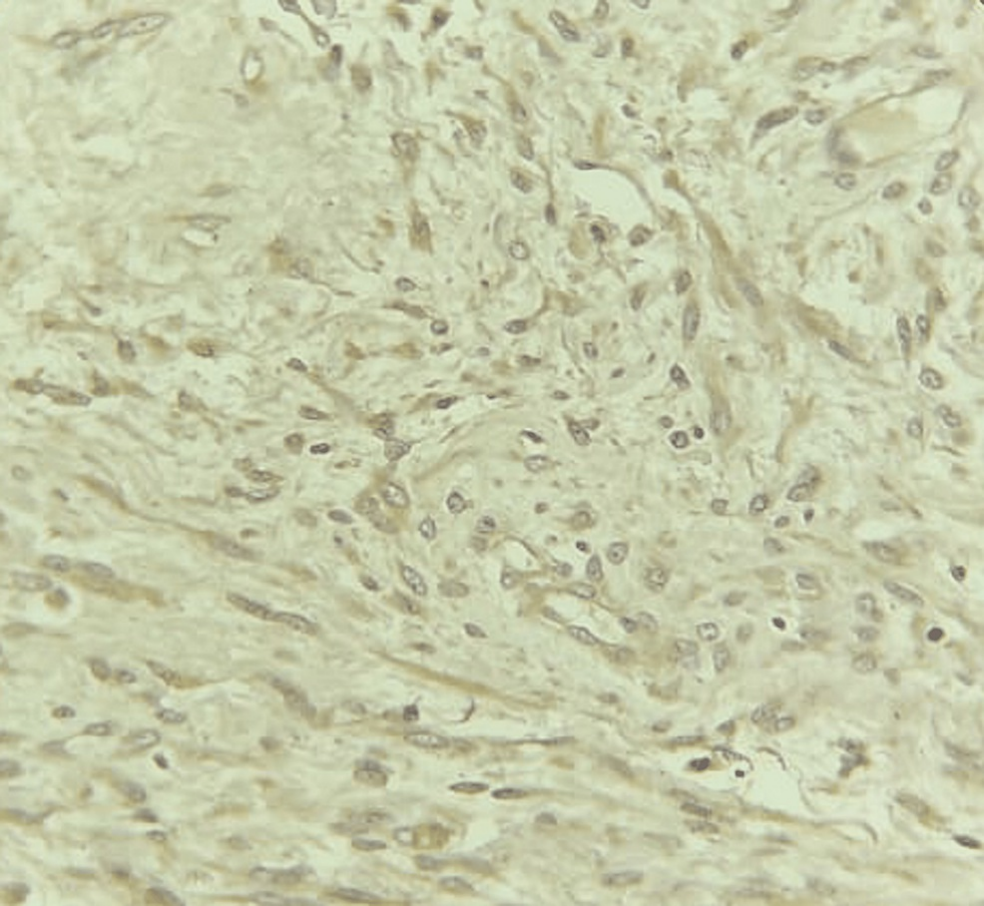
IHC of the specimen showing DOG1 positivity. IHC: Immunohistochemistry; DOG1: Discovered on GIST-1.

On gross examination, a globular, capsulated, greyish-white lobulated mass of size 6 x 4 x 3 cm, with hemorrhagic areas on the cut surface with microscopic findings of polygonal tumor cells arranged in sheets, eosinophilic cytoplasm, eccentric nuclei, and defined nucleoli, was present. The tumor cells were positive for DOG1 and SMA but negative for CD-117, S-100, and CK-20 (Figures 7-8). The features were suggestive of epithelioid E-GIST.

The patient was discharged on the seventh postoperative day with stable vitals. Imatinib therapy was initiated subsequently, and regular follow-up was done every three months for one year. A repeat CT scan of the abdomen and pelvis after a year showed no features of recurrence.

## Discussion

Interstitial cells of Cajal are the mesenchymal cells found in the GI tract, which are responsible for generating spontaneous electrical slow waves that cause smooth muscle contraction resulting in peristalsis and segmentation. GIST arises from the interstitial cells of Cajal. The majority of these tumors have an activating mutation in the KIT proto-oncogene (CD117) or PDGFRA gene mutation. However, the c-KIT mutation is absent in 4-15% of cases, which can complicate the diagnosis of GIST [1]. This led to the discovery of DOG1, which encodes for the protein anoctamin, also called DOG1 protein. DOG1 positivity is considered independent of KIT or PDGFRA mutation status and is considered clinically significant in diagnosing GIST. A study published by Lee CH et al. showed high overall sensitivity and specificity for the DOG1 marker in diagnosing GIST [2]. It was seen that about six percent of tumors with c-KIT negativity were positive for the DOG1 marker [3]. The study also showed that DOG1 antibodies were more sensitive than CD117 antibodies in detecting tumors with PDGFRA mutation, gastric origin, or epitheloid nature. A study conducted by Liegl B et al. also reported DOG1 to have superior sensitivity and specificity compared to C-KIT (CD117) and CD34 [4].

E-GISTs have the same histological features as GISTs but arise from structures other than the GI wall, commonly from the omentum, mesentery, abdominal wall, or retroperitoneum [5]. Most E-GISTs were either GISTs with extensive extramural growth resulting in loss of contact to the external muscle coat of the gut or as a metastasis from an inoperable GIST [6]. It often presents in adulthood with no gender predilection. The diagnosis and management of E-GIST are similar to that of intestinal GISTs. Symptoms vary from mild, dull aching abdominal pain to melena, hematemesis, or cases of frank rupture and hemorrhage into the peritoneal cavity.

In 2013, Cöl C and Yilmaz F published a case report of an E-GIST presenting as a giant abdominopelvic tumor [7]. They resected a 15 x 15 cm abdominopelvic mass, which had no attachment to the stomach, intestines, gall bladder, spleen, or other abdominal or gynecological organs. On histopathological examination, it showed CD117 and vimentin positivity. Santos PO et al. reported an unusual presentation of an E-GIST, where a 51-year-old female presented with a large abdominal lump, which on CT revealed a large intraperitoneal tumor measuring up to 30 cm and compressing the surrounding structures. On laparotomy, the lesion adhered to the greater gastric curvature and the vesical peritoneum. Histopathological examination showed GIST of extra-gastrointestinal location (presumably peritoneal) of mixed subtype [8].

Radiological investigations are done once there is clinical suspicion of a lump. Contrast-enhanced CT scan of the abdomen and pelvis is the investigation of choice for staging and follow-up [8]. MRI and positron emission tomography (PET) can be used for the early detection of tumors for neo-adjuvant therapy. In addition, PET-CT can be used to assess the response to imatinib treatment [9].

The tumor is not classified as malignant or benign but based on its malignant potential into a spectrum of very low-risk to high-risk malignant tumors [8]. Currently, the standard treatment is surgical excision, which involves removing the tumor with intact pseudocapsule and negative microscopic margins [10]. In cases of high-risk malignant tumors, recurrence, or metastasis-specific, tyrosine kinase inhibitors like imatinib mesylate therapy are used. Sunitinib and ponatinib are used for treatment in cases with resistance or partial response to imatinib [10]. Often in non-resectable tumors or those with a high risk of malignant potential, the neoadjuvant treatment is given with tyrosine kinase inhibitors.

Important prognostic factors include operability and mitotic rate [11]. The overall survival rates of E-GIST patients are significantly lower than that of GIST patients, in which first, third, and fifth-year survival rates are 91.7%, 61.1%, and 48.9%, respectively [12].

The two cases studied above presented as slow-growing tumors, which upon doing radiological investigations and biopsy, showed neoplastic growth suspicious for GIST. The growth was excised in both cases and was positive for DOG1 but negative for CD-117 on histopathological examination. Upon which a diagnosis of E-GIST was made. Both patients are being followed up regularly for recurrence and advised for imatinib therapy.

## Conclusions

In patients presenting with large abdominal masses, a differential of E-GIST should be considered. Multi-detector computed tomography (MDCT) and histopathological evaluation are to be used for correct diagnosis and management. The origin of E-GIST is to be further investigated, and more studies are required to evaluate CD117-negative, DOG1-positive E-GIST.
